# The relationship between sleep quality, neck pain, shoulder pain and disability, physical activity, and health perception among middle-aged women: a cross-sectional study

**DOI:** 10.1186/s12905-022-01773-3

**Published:** 2022-05-21

**Authors:** Myung Kyung Lee, Jihyun Oh

**Affiliations:** 1 College of Nursing, Research Institute of Nursing Science, Kyungpook National University, Daegu, 41944 Korea; 2 Department of Nursing, College of Nursing and Health, Kongju National University, Kongju, 32588 Korea

**Keywords:** Sleep quality, Shoulder pain, Women, Health perception, Neck pain

## Abstract

**Background:**

Sleep quality is an important physical requirement for a healthy life, and good sleep quality has been recognized as a significant component in physical and mental health and well-being. The purpose of this study was to identify the factors that affect sleep quality as well as the relationship between sleep quality and neck pain, shoulder pain and disability, physical activity, and health perception.

**Methods:**

We conducted surveys on 494 women between the age of 35 and 64 years. The study evaluated neck pain, shoulder pain and disability, physical activity, self-health perception and sleep quality with self-reported questionnaires in middle-aged women. Data were analyzed using SPSS 23.0.

**Results:**

The results showed that the more severe the neck pain and shoulder pain and disability, the worse the sleep quality was in middle-aged women and the better the health perception, the lower the sleep quality score was, indicating good sleep quality. Shoulder pain, self-perceived task difficulty, and health perception were identified as variables that affected the sleep quality in middle-aged women. The explanatory power of the model in explaining sleep quality was 22.9%.

**Conclusions:**

Worsened shoulder pain, self-perceived task difficulty, and negative health perception can affect poor sleep quality; therefore, it is necessary to develop health interventions for pain management and emotional and social support for improving daily sleep quality. To improve the sleep quality in middle-aged women, healthcare workers should consider the subjects’ pain and functional disability, in accordance with their health perception.

## Background

Chronic neck pain affects 16.7–75.1% of the world’s population annually, and such neck pain is thought to be caused by various factors, including hormonal changes and mechanical and postural overload [1]. Shoulder pain occurs with a lifetime prevalence of 6.7–66.7% [2]. Neck pain, shoulder pain, and shoulder disabilities are the most common illnesses among musculoskeletal disorders that reduce work efficiency as well as work productivity [3, 4]. Workers with a high level of physically demanding work are at a higher risk of developing work-related neck disorders and disabilities than those with a lower level of strenuous work [5, 6]. People with neck pain are more prone to experiencing lower sleep quality than those with no neck pain. In middle-aged women, particular, estrogen levels, and physiological functions gradually decrease with the onset of menopause [7]. Middle-aged women experience discomfort due to female hormonal changes (e.g., menopause) that aggravate sleep disorders that interfere with sleep quality [1, 8]. It was found that neck or shoulder pain and disability can affect the degree of physical exertion at one’s current job while also interfering with sleep quality [8, 9].

Good sleep quality has been recognized as a significant component of physical and mental health and well-being [10]. The subjects’ sleep quality affects not only their quality of life but also their physical, mental, and social aspects [8, 11]. Therefore, examining the factors that affect sleep quality in middle-aged women will have significant ramifications.

The degree of one’s self-perceived health affects sleep quality [12], and the degree of one’s health perception varies according to the changes in physical function that appears with age and the development of chronic illnesses. Therefore, from Rosenstock’s Health education: Theory, research, and practice [13] point of view, which emphasizes that one of the ways to control one’s illness is to have a fixed belief in one’s health. An individual’s belief and attitude toward physical and mental illness are important factors in influencing health behaviors. Poor perceived health is associated with short and long sleep duration, sleep disturbance, and elevated risk of chronic diseases including diabetes mellitus, hypertension, and cardiovascular disease [14–16]. Subjective and self-reported health perception provide a significant predictor of individual health status [17, 18], resulting in substantial health problems. Therefore, it is important to investigate the relationship between health perception and sleep quality.

As one of such health behaviors, regular exercise is the easiest behavior to incorporate into daily life. It not only improves physical health but also affects sleep quality, which is important in maintaining a long-lasting healthy life [8, 19]. Previous studies reported that physical activity showed the lowest score of occurrences among health-promoting behaviors [20] and that working women and elderly women have low sleep quality [21, 22]. However, there are very few studies that have examined the sleep quality and physical activity among middle-aged women in South Korea. Physical activity is known to maintain women’s health, and physical inactivity is an important factor in various chronic diseases such as obesity, diabetes, and cardiovascular diseases [23–25]. In addition, people who spend long periods of time sitting down or being physically inactive tend to have shorter sleep periods or poor sleep quality [19, 26, 27]. Based on previous studies, the level of physical activity is an important factor in deciding sleep quality. However, previous studies have shown that there is no correlation between physical activity and sleep quality [28]. Despite studies that show physical activity can alleviate insomnia symptoms in middle-aged women [29], there are studies that show physical activity has no effect on improving sleep quality. Thus, it is important to examine how physical activity affects sleep in middle-aged women in South Korea. Therefore, this study aimed to investigate the effects of neck pain, shoulder pain and disability, physical activity, and health perception on sleep quality in middle-aged women.

## Methods

### Design and sample

This descriptive research study was designed to identify the relationship between sleep quality and neck pain, shoulder pain and disability, physical activity, and health perception and to analyze factors that affect sleep quality. Shin et al. [30] has reported that “the age of 35 and 64 years” denotes “middle-age” in women in South Korea. Neck and shoulder pain were reported to be more prevalent among middle-aged women than men [31–35]. A convenience sample of 515 middle-aged women between 35 and 64 years, who had visited two community centers located at Y gu in Seoul was drawn from the Seoul metropolitan, which was the target city because Seoul is the largest and the most populated city to be representative of South Korea and one fifth of the South Korean population lives in Seoul.

**Fig. 1:**
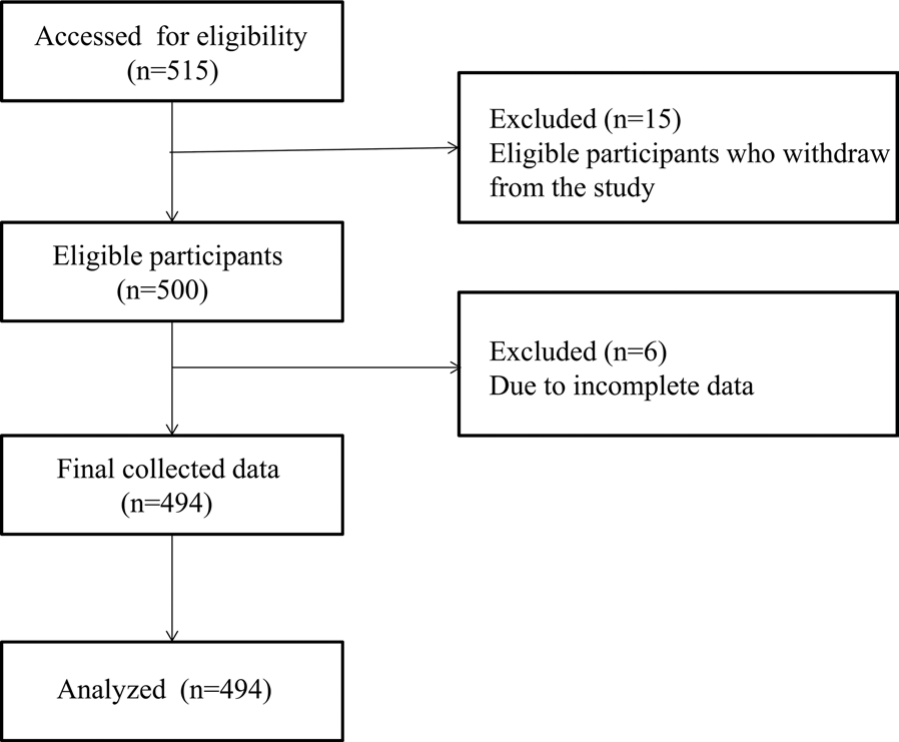
Flow chart of the study

A total of 515 questionnaires were distributed by personal contact exercising appropriate precautions during COVID-19 pandemic. After directly providing sufficient explanation of the questionnaires to the participants, cooperation and consent were obtained as well as the consent for participation in the study. The questionnaires were then distributed, and after having the participants self-report, the completed questionnaires were collected. The time spent to collect the data for each participant was approximately 15 min. Of the 515 questionnaires distributed and collected through this process, 494 were used as the final valid samples, excluding the data that were judged as being incomplete or insincerely answered (Questionnaire response rate: 95.9%) (Fig. 1).

The study was conducted from June 1 to 22, 2021. The inclusion criteria were: (1) being a woman aged 35 to 64 years, and (2) being a woman who understood the purpose and content of the study and agreeing to participate in the study. We have excluded participants who were unable to communicate and have intellectual disability.

### Measurement

The sociodemographic characteristics of the participants included age, marital status, education, smoking, alcohol, economic status, number of comorbidities, perceived physical difficulty of one’s work, level of physical activity, and type of job.

#### Sleep quality

Sleep quality was assessed using the Pittsburgh Sleep Quality Index (PSQI) developed by Buysse, Reynolds III, Monk, Berman, and Kupfer [36], which is a self-report questionnaire comprising 19 items. The PSQI has seven components: subjective sleep quality, sleep latency, total sleep duration, sleep efficiency, sleep disturbance, use of sleep medication, and daytime dysfunction. It is a tool that has been used extensively for a wide range of populations and shows the severity of several sleep problems. The total PSQI score ranges from 0 to 21 with each of the seven components scored from 0 to 3, which are then summed to produce the global score. Buysse [36] defines a score of ≥ 5 as poor sleep and of < 5 as good sleep. Hence, the seven components of PSQI as well as the total PSQI score indicate that the higher the sleep score, the poorer the sleep quality. As for the tool’s reliability, Cronbach’s α for this scale was 0.90 in this study.

#### Shoulder Pain and Disability Index

The Shoulder Pain and Disability Index (SPADI) is a self-report assessment tool that assesses shoulder pain and disability. This assessment tool has two subsections and 13 items in total, with five items in the pain subsection and eight items in the disability subsection. For each of the items in the two subsections, the score ranged from 0 (no pain or difficulty) to 10 (worst pain or disability) [37]. The final score is calculated by summing the total pain score and total disability score with 100 as the full score. A higher score in each subsection means more severe the pain or higher level of disability [38]. The Cronbach’s α in this study was 0.96.

#### Northwick Park Neck Pain Questionnaire

The Northwick Park Neck Pain Questionnaire (NPQ) [39] is a self-report assessment tool that consists of nine daily activity-related items (intensity, sleep, numbness, duration, carrying things, reading/watching television, working, social activities, and driving) that are affected by neck pain. Each item consists of one question and five responses that indicate increasing level of difficulty and pain. The patient chooses one response that is the most reflective of their current situation. Each item ranges from a score of 0 to 4 with 4 indicating the worst disability. The total score is the summation of scores from the nine items (range of possible scores: 0–36). In this study, a Korean version of the NPQ translated and verified for reliability and validity by Lee et al. [40] was used, and the Cronbach’s ⍺ in this study was 0.93.

#### Health perception

Health perception refers to the state of an individual’s perception that governs their health behavior [41]. In this study, the scale for measuring general health perception developed by Ware [41] was translated to suit Koreans, and the assessment tool modified by Lee [42] was used. This assessment tool has a total of 20 items and consists of current health, prior health, health outlook, health worries and concerns, resistance and susceptibility, and rejection of sick role as components. It is on a 4-point Likert scale, and a higher score indicates a higher subjective health status. The Cronbach’s α in this study was 0.86.

#### International Physical Activity Questionnaire

To measure the subjective physical activity in this study, a Korean version of the International Physical Activity Questionnaire (IPAQ) short form was used, which is an assessment tool used to evaluate daily physical activity comprehensively and objectively [43]. IPAQ short form assesses the frequency and the average duration per day at which > 10 min of vigorous physical activity (VPA), moderate physical activity (MPA), walking, and sitting was performed within the frame of last 7 days. In this study, six questions were used to determine the overall average metabolic equivalent (MET) score when calculating the metabolic equivalent unit (Metabolic Equivalent Unit: MET-min/week), excluding the first question which asks about “time spent sitting” (Walking = 3.3 METs, Moderate Physical Activity = 4.0 METs and Vigorous Physical Activity = 8.0 METs). The amount of physical activity was converted into a metabolic equivalent unit according to the IPAQ scoring protocol, and the physical activity of the participants was then categorized into inactive, minimally active or health-enhancing physical activity. “Inactive” is the least amount of physical activity and refers to individuals who do not meet the criteria for either minimally active or health-enhancing physical activity. “Minimally active” category refers to those who meet any of the following criteria: ≥ 3 days of VPA of at least 20 min per day, ≥ 5 days of MPA and/or walking of at least 30 min per day, and lastly, ≥ 5 days of any combination of walking, MPA, and VPA, achieving a minimum total physical activity of 600 MET-min/week. “Health-enhancing physical activity” is the most ideal category of physical activity and refers to those who meet either of the following criteria: VPA on at least 3 days in the last 7 days, achieving a minimum of total physical activity of 1,500 MET-min/week or ≥ 7 days of any combination of walking, MPA or VPA, achieving a minimum of total physical activity of 3,000 MET-min/week.

### Ethical considerations

This study was approved by the Institutional Review Board at the Daejeon University (1040647-202103-HR-006-02) to secure research ethics for the participants. The participants provided informed written consent after they were given an explanation about the purpose of the study, their freedom to withdraw from the study at any point, the confidentiality of the data collected in accordance with the Personal Information Protection Act, the strict research-only usage of the data collected, anonymization of the data, and the document’s safekeeping in a location with a double lock. The copy of the explanations and the consent form was provided to the participants, and the completed questionnaires and consent form were collected and kept in individual files to protect personal information. The research was performed in accordance with the ethical guidelines of the Declaration of Helsinki.

### Statistical analyses

All data collected were analyzed using SPSS version 23.0 (IBM Corp., Armonk, NY, USA). The participants’ general characteristics, neck pain, shoulder pain and disability, physical activity, health perception, and sleep quality were calculated using the mean and standard deviation for continuous variables, and frequency and percentage for categorical variables. The difference in sleep quality in accordance with the participants’ general characteristics was analyzed using independent t-tests and a one-way analysis of variance (ANOVA) for categorical variables and the post hoc analysis was verified using Sheffé test. The correlation between the participants’ neck pain, shoulder pain and disability, physical activity, and health perception and sleep quality was analyzed using Pearson’s correlation coefficient. Stepwise multiple regression was used to analyze the degree of effect on the participant’s sleep quality after testing for multicollinearity. A statistically significant level was a p-value < 0.05.

## Results

### Participants’ general characteristics and differences

Of the 515 participants, 494 participants’ responses were analyzed for the final data, excluding insincere and omitted responses (response rate: 95.9%). The average age of the participants was 42.40 (6.51) years, and the range was 35–63 years. Regarding marital status, 357 (72.3%) participants were married, which was more than half of the participants, and 400 (81.0%) participants had received a college degree. Regarding drinking and smoking, 479 (97.0%) participants were nonsmokers and 271 (54.9%) did not drink alcohol at all; 390 (78.9%) answered that their economic status was “middle-income,” and 320 (64.8%) participants did not have any comorbidities. Furthermore, 407 (82.4%) participants had a job and when asked about their perceived degree of difficulty of their work, 245 (49.6%) responded that it was moderate. Lastly, in analyzing the ratio according to the level of physical activity, 294 (52.6%) participants were inactive, 167 (29.9%) were minimally active, and 33 (5.9%) were active in pursuing health-enhancing physical activities (Table 1).

**Table 1:** Descriptive statistics (*N* = 494)

Characteristics	Mean (SD)	Range	n (%)	PSQI global score	
				Mean	t or F (*p*) Scheffé
Age (year)	42.40 (6.51)	35–63			
≥ 35, ≤ 39^a^			209 (42.3)	9.31 (2.63)	4.635 (0.010)
≥ 40, ≤ 49^b^			201 (40.7)	8.60 (2.74)	a > b
≥ 50^c^			84 (17.0)	8.47 (2.75)	
Marital status					
Single			125 (23.3)	8.61 (2.74)	1.313 (0.270)
Married			357 (72.3)	8.94 (2.70)	
Divorced & widowed			12 (2.4)	9.75 (2.76)	
Education					
≤ Middle school			94 (19.0)	9.12 (2.75)	0.971 (0.332)
College			400 (81.0)	8.82 (2.70)	
Smoking					
Yes			15 (3.0)	9.40 (2.82)	0.748 (0.455)
No			479 (97.0)	8.86 (2.71)	
Alcohol					
Yes			223 (45.1)	9.06 (2.62)	1.371 (0.171)
No			271 (54.9)	8.73 (2.78)	
Economic status					
High			25 (5.1)	8.92 (2.59)	0.003 (0.997)
Middle			390 (78.9)	8.88 (2.73)	
Low			79 (16.0)	8.87 (2.68)	
No. of comorbidities					
0			320 (64.8)	8.68 (2.68)	3.093 (0.046)
1			154 (31.2)	9.16 (2.80)	
≥ 2			20 (4.0)	9.90 (2.33)	
Perceived physical difficulty of one’s work					
Easy^a^			96 (19.4)	8.02 (2.56)	17.650 (< 0.001)
Moderate^b^			245 (49.6)	8.59 (2.59)	a, b < c
Difficult^c^			153 (31.0)	9.88 (2.72)	
Level of physical activity					
Inactive			294 (52.6)	8.99 (2.75)	0.690 (0.502)
Minimally active			167 (29.9)	8.74 (2.67)	
HEPA-active			33 (5.9)	8.57 (2.62)	
Job					
Employed (partly or full time)			407 (82.4)	8.88 (2.73)	0.077 (0.938)
Unemployed			87 (17.6)	8.86 (2.64)	

HEPA, health-enhancing physical activity

The results of the analysis of the total PSQI score in accordance with the general characteristics showed that there were statistically significant differences depending on the age (F = 4.635, p < 0.001), number of comorbidities (F = 3.093, p = 0.046), and perceived physical difficulty of one’s work (F = 17.650, p < 0.001). Regarding the participants’ age, post hoc analysis using the Scheffé test showed that participants aged 35–39 years had a statistically significantly worse sleep quality than those in their 40 s. A statistically significant difference showed in sleep quality according to the number of comorbidities. Those with ≥ 2 comorbid diseases had a higher sleep quality score than those with none. A post hoc analysis using the Scheffé test showed no difference. There was a statistically significant difference in sleep quality according to the perceived physical difficulty of one’s work, and a post hoc analysis using the Scheffé test showed that participants who said their work was physically difficult had the worst sleep quality.

### Scores for NPQ, SPADI, physical activity, health perception, and sleep quality

As shown in Table 2, the total PSQI average score was 8.88 (2.71), ranging from 2 to 17 points. Within PSQI’s components, the average of subjective sleep quality was 1.31 (0.63), of sleep latency was 1.38 (0.90), of sleep duration was 0.47 (0.73), of habitual sleep efficiency was 0.56 (0.98), of sleep disturbances were 1.25 (0.56), of sleeping medicine usage was 0.18 (0.59) and of daytime dysfunction was 1.24 (0.94). As for sleep quality, the average of poor sleep quality was 9.42 (2.33) and good sleep quality was 4.29 (0.87). As a result of analyzing the level of physical activity, the total average of the IPAQ was 1723.3 min per week, walking activity was 785.15 min per week, moderate activity was 354.45 min per week, and vigorous activity was 577.00 min per week. As for shoulder pain and disability, the average for shoulder pain was 42.55 (24.72) out of 100points and shoulder disability was 27.29(23.38) out of 100 points. The average of neck pain was 31.51 (19.42) and 49.27 (7.03) for health perception.

**Table 2:** Level of NPQ, SPADI, IPAQ, Health perception, and PSQI (*N* = 494)

Variables	Min–max	Mean (SD)	n (%)
Total PSQI score	2–17	8.88 (2.71)	
Subjective sleep quality	0–3	1.31 (0.63)	
Sleep latency	0–3	1.38 (0.90)	
Sleep duration	0–3	0.47 (0.73)	
Habitual sleep efficiency	0–3	0.56 (0.98)	
Sleep disturbances	0–3	1.27 (0.56)	
Use of sleeping medicine	0–3	0.18 (0.59)	
Daytime dysfunction	0–3	1.24 (0.94)	
Good sleep quality		4.29 (0.87)	51 (10.3)
Poor sleep quality		9.41 (2.33)	443 (89.7)
IPAQ score, min/week	0–39,680	1723.30 (2824.31)	
Vigorous activity, min/week	0–26,880	577.00 (1931.42)	
Moderate activity, min/week	0–12,800	354.45 (1056.30)	
Walking activity, min/week	0–8316	785.15 (1049.19)	
SPADI	0–89.38	33.95 (21.90)	
Pain subscale	0–98	42.55 (24.72)	
Disability subscale	0–100	27.29 (23.38)	
NPQ	0–83.33	31.51 (19.42)	
Health perception	20–76	49.27 (7.03)	

NPQ, Northwick Park Neck Pain Questionnaire; SPADI, Shoulder Pain and Disability Index; IPAQ, International Physical Activity Questionnaire; PSQI, Pittsburgh Sleep Quality Index

### Correlations between NPQ, SPADI, physical activity, health perception, and sleep quality

The correlation between variables is shown in Table 3. Neck pain had a positive correlation with SPADI’s components, pain (r = 0.418, p < 0.001) and disability (r = 0.404, p < 0.001), as well as total sleep quality (r = 0.253, p < 0.001), but showed a negative correlation with health perception (r =  − 0.326, p < 0.001). Among SPADI’s components, pain showed a positive correlation with disability (r = 0.708, p < 0.001) and total sleep quality (r = 0.404, p = 0.004), but a negative correlation with health perception (r =  − 0.347, p < 0.001). Among SPADI’s components, disability showed a positive correlation with sleep quality (r = 0.301, p < 0.001) and a negative correlation with health perception (r =  − 0.302, p < 0.001). Moreover, sleep quality and health perception showed a negative correlation (r =  − 0.369, p < 0.001).

**Table 3:** Correlations among study variables (*N* = 494)

Variables	NPQ	SPADI pain subscale	SPADI disability subscale	IPAQ	PSQI	Health perception
NPQ	–					
SPADI pain subscale	0.418 (< 0.001)	–				
SPADI disability subscale	0.404 (< 0.001)	0.708 (< 0.001)	–			
IPAQ	0.044 (0.329)	0.041 (0.361)	0.041 (0.386)	–		
PSQI	0.253 (< 0.001)	0.404 (0.004)	0.301 (< 0.001)	0.001 (0.977)	–	
Health perception	− 0.326 (< 0.001)	− 0.347 (< 0.001)	− 0.302 (< 0.001)	0.057 (0.208)	− 0.369 (< 0.001)	–

NPQ, Northwick park neck pain questionnaire; SPADI, Shoulder Pain and Disability Index; PSQI, Pittsburgh Sleep Quality Index; IPAQ, International Physical Activity Questionnaire

### Influential factors of sleep quality

To investigate the influencing factors of sleep quality among middle-aged women, prior to the stepwise multiple regression analyses, the relationships between independent variables (e.g., neck pain, shoulder pain and disability, health perception, and sociodemographic characteristics) and the dependent variables (e.g., sleep quality) were examined. Table 4 shows that neck pain, shoulder pain and disability, health perception, physical activity, and particular sociodemographic characteristics were significantly different in relation to sleep quality: for instance, age, number of comorbidities. Moreover, perceived physical difficulty of one’s work were analyzed with stepwise multiple regression analyses. Factors that affected sleep quality were pain of SPADI (β = 0.293, p < 0.001), perceived physical difficulty of one’s work (β = 0.111, p = 0.009), and health perception (β =  − 0.248, p < 0.001). The explanatory power of the model in explaining sleep quality was 22.9% (Table 4). These results show that the more severe the shoulder pain and the more difficulty one perceives from their work, the worse the sleep quality and that the better the health perception, the better the sleep quality.

**Table 4:** Results of the stepwise multiple regression analysis for PSQI (*N* = 494)

Variables	PSQI				
	β (SE)	*95% CI*		*t*	*p*
Constant	11.487 (1.051)	9.422	13.551	10.392	< 0.001
SPADI pain subscale	0.293 (0.005)	0.023	0.042	6.634	< 0.001
Perceived physical difficulty of one’s work	0.111 (0.165)	0.108	0.757	2.621	0.009
Health perception	− 0.248 (0.017)	− 0.128	− 0.062	− 5.637	< 0.001
Adjusted R^2^	0.229				

CI, Confidence Interval

## Discussion

This study aimed to provide fundamental information for healthcare interventions that can improve sleep quality in middle-aged women by identifying the factors that affect sleep quality as well as the correlation between sleep quality and neck pain, shoulder pain and disability, physical activity, and health perception.

This study showed statistically significant differences in sleep quality in relation to the general characteristics of middle-aged women, age, number of comorbidities, and the perceived physical work difficulty. The findings of this study suggest that sleep quality is worse with younger age. Among the participants, those who were employed accounted for the most at 82.4%, and most women in their 30 s had both home and wok responsibilities. Therefore, occupational stress and the physical burden of housework and childcare negatively impact sleep quality. For this reason, planned support measures for these women are necessary by extensively assessing the occupational, environmental, and situational factors. This study found that the more comorbid diseases participants had, the worse was their sleep quality when compared to those without comorbid diseases. This finding corroborates with various preceding studies [22, 44–46]. Patients with chronic lower back pain [45] and diabetes reportedly experience decreased sleep quality and sleep efficiency [44]. In particular, reports of decreased sleep quality in patients with diabetes are associated with symptoms such as nocturia, nocturnal hypoglycemia, and obstructive sleep apnea [46]. In older persons, a higher number of chronic and comorbid diseases is associated with a higher rate of sleep disturbances and lower sleep efficiency due to persistent pain and disability. Additionally, lower sleep quality and quality of life lead to waking up at dawn due to night pain or struggling to fall asleep again [22, 47].

This study has limitations in that there was no comprehensive analysis of sleep quality in relation to the type of illness. Thus, it was found that sleep quality for participants with many comorbidities are poor. Therefore, physiological health problems such as comorbidities in middle-aged women are thought to affect sleep quality directly or indirectly, which indicates that health interventions can improve sleep quality and quality of life; 31% of participants reported that their present perception of physical burden from their work is high and their sleep quality appeared to be the worst. The burden of housework and childcare related to women without jobs, for those with jobs, could be stem from work difficulty, job stress, and the burden of housework and childcare. This is consistent with a previous study in which, compared with men, women experience burden accompanied by various changes, such as housework, childcare, work, child education, as well as hormonal changes in middle-aged women, which negatively effects sleep quality [48].

The average score of the participants of this study showed that they were poor sleepers, which is similar to the findings of other studies [29, 49], that examined sleep quality in middle-aged women and obtained a score of 8.4 [29, 49] but is higher than the score of 7.76 obtained in one of the studies [50]. This result implies that sleep disorders among South Korean middle-aged women are common and they tend to ignore their sleep management. This could be attributed to their dismissing it as a menopausal symptom experienced in midlife or because of neglecting one’s health by caring only for the health of the family and sacrificing for the family despite their own poor health conditions subjectively felt due to the burden of childcare, housework and workplace stress.

In addition, this study showed a positive correlation between sleep quality and neck pain and shoulder pain and disability and a negative correlation between sleep quality and health perception. As such, the more severe the neck pain and shoulder pain and disability, the higher the score for sleep quality, demonstrating a poor quality of sleep. The higher the self-health perception was, the lower was the sleep quality score, hence demonstrating good sleep quality. This corroborates with previous studies [51, 52] that show the more severe the neck pain, the more severe the shoulder pain, and the more severe the shoulder pain, the more severe the shoulder disability and that such symptoms or disabilities worsen sleep quality [53]. Women complain of chronic pain as they spend more time making repetitive movements in the same posture for a long time due to work or house chores. Since these pains negatively affect sleep quality due to the accompanying muscle dysfunction, it seems necessary to establish an intervention that can fundamentally improve health promotion through investigating their lifestyle and postures in daily life according to the area of chronic pain.

In this study, shoulder pain, perceived difficulty of one’s work, and health perception were significant factors affecting sleep quality in middle-aged women. Similar results were obtained in a study by Bintang et al. [54] and Lee and Oh [22] in which pain affected sleep quality and a more severe pain resulted in a higher sleep quality score, supporting this study’s findings. Moreover, the perceived difficulty of one’s work can be affected by various factors (e.g., occupation, house chores, childcare and health concerns), which can consequently affect one’s health perception and further worsen sleep quality [41]. This study found no correlation between sleep quality and physical activity that corresponds with previous studies that also found that the level of women’s physical activity had no impact on sleep quality [55]. Specifically, this study found that the participants’ physiological symptoms, physical burden and health perceptions were more important factors in sleep quality than physical activity.

This study has some limitations. First, the subjects of the study were South Korean middle-aged women residing in Seoul. Moreover, this study was conducted by convenience sampling of middle-aged women in a small setting; hence, the results cannot be generalized to other populations. Second, because the sleep quality in middle-aged women was assessed using self-report tools, it may not be accurate in identifying the actual sleep pattern. Finally, this study is a cross-sectional study; hence, it is limited in that the cause and effect between variables cannot be analyzed in various ways. Despite these limitations, considering the study’s findings, middle-aged women’s shoulder pain, perceived work difficulty and health perception were found to be important factors in determining sleep quality. These results suggest that the severe physical symptoms of shoulder pain of which middle-aged women complain can negatively affect sleep quality; therefore, considering these physical symptoms, interventions to improve sleep quality are needed. Further, the results of this study show that healthcare workers should consider the subjects’ health and other symptoms or pain to improve sleep quality in middle-aged women. Importantly, perceived difficulty of one’s job and health can affect sleep quality necessitating the development of a health intervention strategy to provide physical healthcare, and mental and social support to lessen the burden off middle-aged women. In a follow-up study, healthcare providers in clinical practice should plan and provide health education and healthcare interventions that enhance sleep quality in middle-aged women to ultimately improve their physical and mental quality of life, based on the results of previous studies that show physical symptoms affect sleep quality that affects individual physiological and mental health [22, 56] and the results of this study. This should be done based on objectively measured data of the sleeping state, while considering their physiological symptoms in relation to their comorbidities and age.

## Conclusions

The novelty of this study is that it identified the significant correlations between sleep quality and neck pain, shoulder pain and disability, physical activity, and health perception. The factors causing poor sleep quality were shoulder pain, physical difficulties in one’s work, and poor health perception, which explained sleep quality as a 22.9% explanatory power. These results suggest the following: Sleep education and health intervention are needed to improve sleep quality by assessing the shoulder pain and physical difficulties in one’s work based on middle-aged women’s perception of physical health.

## Data Availability

The data presented in this study are available on request from the corresponding author. The data are not publicly available owing to privacy.

## Abbreviations

IPAQ: International Physical Activity Questionnaire
MPA: Moderate physical activity
NPQ: Neck Pain Questionnaire
PSQI: Pittsburgh Sleep Quality Index
SPADI: Shoulder Pain and Disability Index
VPA: Vigorous physical activity
